# Preliminary results indicate that regular training induces high protection against oxidative stress in basketball players compared to soccer

**DOI:** 10.1038/s41598-022-23351-1

**Published:** 2022-11-02

**Authors:** Simone Luti, Rosamaria Militello, Tania Fiaschi, Francesca Magherini, Tania Gamberi, Matteo Parri, Riccardo Marzocchini, Simone Pratesi, Riccardo Soldaini, Alessandra Modesti, Pietro A. Modesti

**Affiliations:** 1 Department of Biomedical, Experimental and Clinical Sciences “Mario Serio”, University of Florence, 50134 Florence, Italy; 2 Department of Experimental and Clinical Medicine, University of Florence, 50134 Florence, Italy

**Keywords:** Biochemistry, Chemical biology, Health care

## Abstract

In elite athlete several metabolic changes occur during regular training. These modifications are associated with changes in blood metabolic profile and can lead to adaptive mechanisms aimed at establish a new dynamic equilibrium, which guarantees better performance. The goal of this study was to characterize the plasma metabolic profile and redox homeostasis, in athletes practicing two different team sports such as soccer and basketball in order to identify potential metabolic pathways underlying the differences in training programs. A cohort of 30 male, 20 professional players (10 soccer and 10 basketballs) and 10 sedentary males as control were enrolled in the study. Plasma redox balance, metabolites and adiponectin were determined. The results show low levels of oxidative species (25.5%), with both high antioxidant capacity (17.6%) and adiponectin level (64.4%) in plasma from basketball players, in comparison to soccer players. Metabolic analysis indicates in basketball players a significant high plasma level of amino acids Valine and Ornithine both involved in redox homeostasis and anti-inflammatory metabolism.

## Introduction

Measurement and monitoring of recovery and fatigue during training periods contribute to the correct method to improve sports performance^1^. When the balance between intensive training and recovery time is not respected, athletes can experience a drop in sports efficiency that is often not recoverable. This can lead to overreaching, and if not recoverable after few weeks or months, it can progress to overtraining syndrome^2^ (OTS) with tipical hormonal, oxidative and metabolic signals^3^. Skeletal muscle is the principal contributors to the exercise-induced reactive oxygen species (ROS) production that can be considered signal molecules^4^ since they regulate the expression of antioxidant enzymes inducing stimulation of the antioxidant defense system and muscle regeneration^5^. However, prolonged strenuous exercise could predispose to muscle damage and lead to a modification of redox homeostasis parameters and to a cascade of metabolic signals that reduce physical performance^6^. Therefore, final beneficial/adverse exercise consequences are the result of the balance between the oxidative stress/inflammation induced by exercise that increases performance and health (hormesis). Chronic training stress causes variations in several metabolic hormones connected to inflammation^7,8^ and among them, adiponectin, an anti-inflammatory hormone secreted in large quantities by adipose tissue^9^ as well as by skeletal muscle^10^, has been observed toincrease in well-trained athletes^11^. This suggests that training could affect the adiponectin response and in sight of this, it is interesting to evaluate the effects of different training protocols on several peripheral signals including adiponectin itself^12^. Metabolomics technologies are useful to understand the athlete’s conditions, to identify metabolic biomarkers, to understand their physical performance and skeletal muscle metabolism^13^. In ball games, such as soccer and basketball, athletes experience muscle stress due to the explosive, stop-and-go nature of these sports. The ability to move at high speed or change direction quickly are specific skills required by both of these sports^14^. Energy systems used in metabolism of these athletes is very similar and is predominantly anaerobic/aerobic alternate although in basketball, the aerobic demand is less than in soccer^15^. The aim of this study is to measure the biochemical internal environment and redox homeostasis in elite soccer and basketball athletes exposed to continuous training sessions over time and to provide an overview of their prevalence comparing the two sports.

## Results

### Participants’ characteristics

Participants’ characteristics are reported in Table 1, in detail mean weight was 81.5 ± 10.2 kg and height was 186 ± 6 cm for basketball players; for soccer players mean weight was 72.7 ± 8.7 kg and height was 180.3 ± 7 cm while for controls mean weight was 73 ± 8.7 and height was 178.7 ± 6. Basketball players were significantly weighed (p-value = 0.03) than soccer players and taller (p-value = 0.03) than controls. Despite all no significant differences were found between body mass indexes (BMI) of participants of the three groups. Controls were elderly than athletes, but the males aged were between 20 and 30 therefore we considered only young males.

**Table 1:** Participants’ characteristics.

Characteristics	Mean^a^ (SD)			Tukey’s test^b^		
	Control	Basketball players	Soccer players	C vs. B	C vs. S	B vs. S
Age (year)	26.1 ± 4.1	21 ± 2.2	20.65 ± 1.1	****	****	0.92
Weight (kg)	73 ± 8.7	81.5 ± 10.2	72.7 ± 8.7	0.09	1	0.03*
Height (cm)	178.7 ± 6	186 ± 6	180.3 ± 7	0.03*	0.79	0.05
BMI (kg/m2)	22.9 ± 2.9	23.6 ± 2.7	22.3 ± 1.5	0.78	0.77	0.29

^a^Measurements’ mean of controls (C), basketball players (B) and soccer players (S); *BMI* = body mass index.

^b^Tukey’s test was performed by Graphpad Prism 6 (*p-value < 0.05), (****p-value < 0.0001).

### Plasma oxidative stress measurements

**Figure 1:**
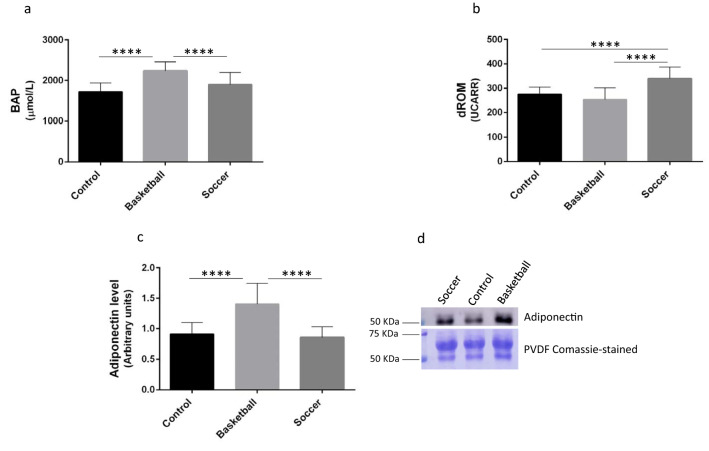
Plasma oxidative stress and Adiponectin determination. (**a**) Antioxidant capacity was measured by BAP Test, (**b**) levels of reactive oxygen metabolites were measured through d-ROM test by a free radical analyzer system, (**c**) Adiponectin level was assessed in 10 µg of proteins from plasma by western blot with adiponectin antibody normalized on total plasma protein content (as reported in methods), (**d**) original western blot from the protein sample All measurements were performed in triplicate and are re-ported in the histograms as mean ± SD. The statistical analysis was carried out by ANOVA Tukey’s test was performed by Graphpad Prism 6 (* p-value < 0.05), (****p-value < 0.0001).

Antioxidant capacity (BAP) and the levels of oxidative species (dROM) are reported in Fig. 1. We noticed that in BAP and dROM values (Fig. 1a,b respectively) significant differences are shown among the three groups. In particular assessing the antioxidant capacity (BAP value), basketball players show a significant increase in comparison to control group (30%; p-value < 0.0001) and soccer players (17.6%; p-value < 0.001) while no significant differences were observed between soccer and control groups. The BAP mean value was 1720.33 ± 216 µmol/L for control group, 2237.17 ± 221 µmol/L for basketball players and 1901.58 ± 294 µmol/L for soccer players. Moreover, no significant differences were observed between the levels of oxidative species (dROM) of basketball players and control; where the mean values were 275.5 ± 29 UCarr for control group and 252.8 ± 48 for basketball players. Soccer players showed an increase of the levels of oxidative species of about 23.2% (p-value < 0.0001) comparing to control group and 34.3% (p-value < 0.001) comparing to basketball players, mean value was 339.4 ± 47 UCarr for soccer players.

### Plasma adiponectin determination

Plasma adiponectin level was determined by western blot in both athletes and healthy controls, and the results are reported in Fig. 1c. The statistical analysis demonstrated that basketball players had a higher level of plasma adiponectin than control group (54.3%; p-value < 0.001) and soccer players (64.4%; p-value < 0.001) while there are not significant differences between soccer and control group.

### Metabolomic analysis using Gas Chromatography/Mass Spectrometry (GC/MS)

**Figure 2:**
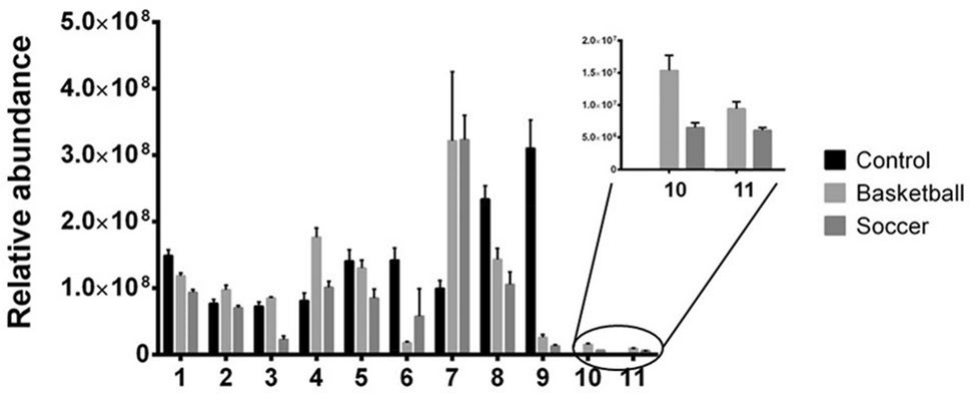
Plasma metabolomic profile using GC–MS analysis. Histogram representation of plasma metabolites whose relative abundance is statistically different among control, soccer and basketball groups, all p-value details are re-ported in Table 2. Numbers of abscissa axis correspond to metabolites as reported in Table 2. All the measurements were performed in triplicate and are reported in the histograms as mean ± SD.

The metabolic profile of plasma from athletes and controls was characterized through GC–MS and each compound obtained was identified by Fiehn library that allowed to identify 11 plasma compounds differentially expressed among the three groups and reported in Table 2. Among them, five have a statistically different concentration in athletes respect to controls. In detail lactic acid (B = 322%, p-value = 0.013; S = 323%, p-value = 0.024) increases in athletes while glutamic acid (B = 79%, p-value = 0.006; S = 62%, p-value = 0.0003), urea (B = 8%, p-value < 0.0001; S = 4%, p-value < 0.0001) and uric acid (B = 61%, p-value = 0.011; S = 45%, p-value = 0.002) show a significant decrease. Moreover, we detected isoleucine and tyrosine only in athletes while these amino acids in our experimental conditions are not measurable in plasma controls (Fig. 2; Table 2). Amino acid valine (217%, p-value = 0.001) and ornithine (120%, p-value = 0.416) show a significant increase only in plasma from basketball players respect to controls and on the contrary glycine (12%, p-value = 0.020) is reduced in the same athletes. Amino acid serine (60%, p-value = 0.026) is reduced in soccer players in comparison to controls. Furthermore, we analyzed metabolites that showed in plasma a statistically significant difference between athletes practicing the two sports and we noticed that most of the differences are related to amino acids. In particular as shown in Fig. 2 and Table 2, in basketball players the following amino acids are statistically increased: l-isoleucine (236%; p-value = 0.003), l-glutamic acid (168%; p-value = 0.011), l-ornithine (138%; p-value = 0.024), l-proline (370%; p-value = 0.0003), l-valine (174%; p-value = 0.010) and l-tyrosine (154%; p-value < 0.007). The same upward trend is shown for Urea (198%; p-value = 0.049).

**Table 2:** List of Metabolites identified in plasma of controls, basketball and soccer players by Gas Chromatography–mass spectrometry (GC–MS) analysis.

N°	Name	Retention Time	CAS number^a^	KEGG ID°	Mean^b^ (SD)			Tukey’s test^c^/Fold change^d^		
					Control	Basketball	Soccer	B vs C	S vs. C	B vs. S
1	l-glutamic acid	[13.232]	56-86-0	C00025	15 ± 0.9	12 ± 0.7	9.4 ± 0.8	**/− 1.2	***/− 1.6	*/ + 1.3
2	l-ornithine	[16.632]	70-26-8	C00077	7 ± 0.6	9.8 ± 1.2	7.1 ± 0.5	*/ + 1.2	ns	*/ + 1.4
3	l-proline	[10.321]	147-85-3	C00148	7.3 ± 0.6	8.5 ± 0.3	2.3 ± 0.9	ns	***/− 3.2	****/ + 3.7
4	l-valine	[9.151]	72-18-4	C00183	8.1 ± 1.1	18 ± 2.4	10 ± 1.5	**/ + 2.2	ns	**/ + 1.8
5	l-serine	[11.174]	56-45-1	C00065	14 ± 1.7	13 ± 2	8.5 ± 2.3	ns	*/− 1.6	ns
6	Glycine	[10.456]	56-40-6	C00037	14 ± 1.8	1.8 ± 0.3	5.8 ± 7.1	*/− 7.8	ns	ns
7	l-( +) lactic acid	[6.851]	79-33-4	C00186	10 ± 1.2	32 ± 18	32 ± 6.4	*/ + 3.2	*/ + 3.2	ns
8	Uric acid	[19.331]	66-22-8	C00366	23 ± 2	14 ± 2.8	11 ± 3.2	*/− 1.6	**/− 2	ns
9	Urea	[9.599]	57-13-6	C00086	31 ± 4.2	2.6 ± 0.7	1.3 ± 0.3	****/− 11.9	****/− 23.8	*/ + 2
10	DL-isoleucine	[10.225]	443-79-8	C00407	nd	1.5 ± 0.2	0.6 ± 0.07	nd	nd	**/ + 2.5
11	Tyrosine	[17.871]	60-18-4	C00082	nd	0.9 ± 0.1	0.6 ± 0.04	nd	nd	**/ + 2

^a^Chemical Abstract Service number KEGG identifier (https://www.genome.jp/kegg/).

^b^The mean of peak area and the respective SD were obtained by GraphPad Prism 6.0 software using the peak area calculated by Agilent MassHunter Quantitative Analysis B08.00 software. All data were reported in order of magnitude 10–7 (nd = not detectable).

^c^Tukey’s post-hoc test was performed by GraphPad Prism 6.0 software reporting the adjusted p-value (*p-value < 0.05), (**p-value < 0.01), (***p-value < 0.001), (****p-value < 0.0001), (ns = not significant).

^d^Fold change was calculated by GraphPad Prism 6.0 software. It is the ratio of the mean of controls (C), basketball players (B) and soccer players (S).

### Metabolomic interaction network

**Figure 3:**
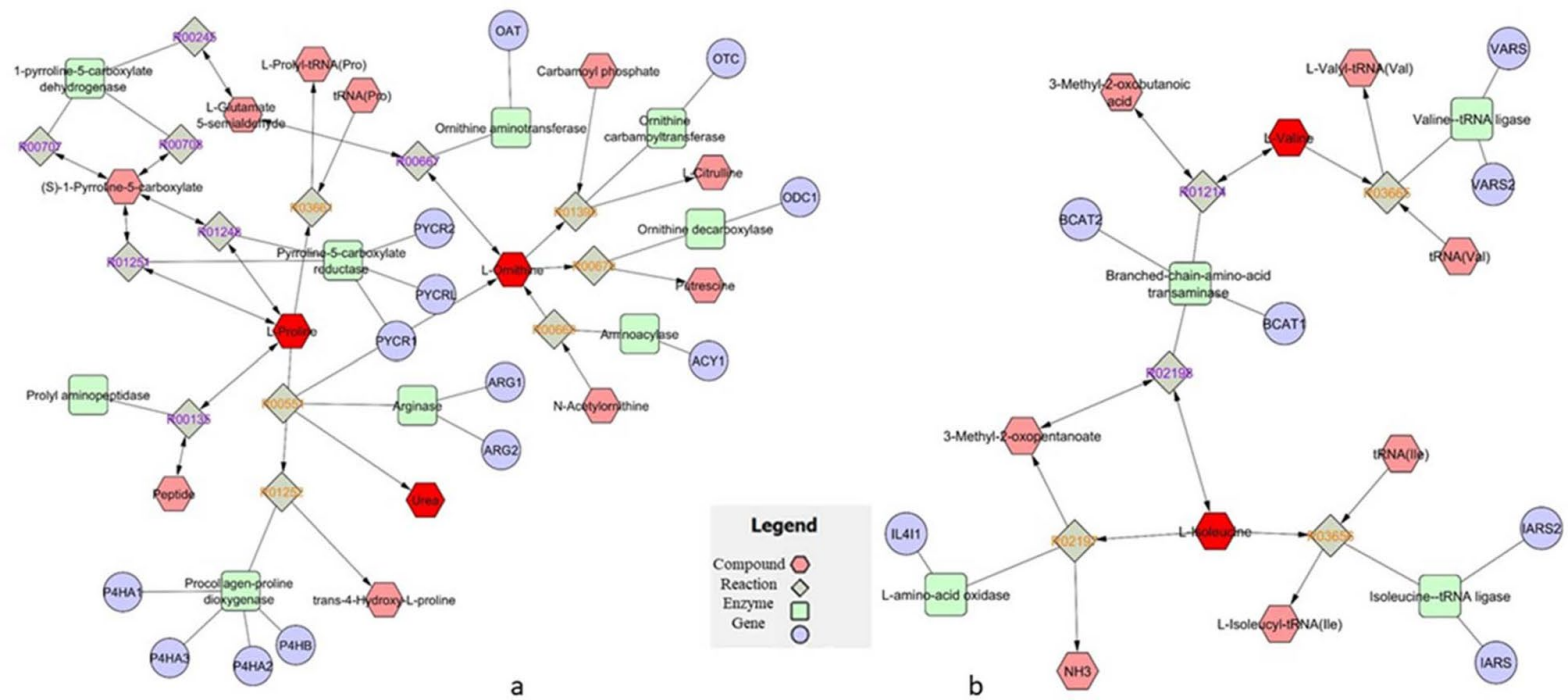
Metabolomics Network interaction analysis on plasma metabolites identified by GC–MS. Selected interaction network of plasma metabolites (see Table 2). The network analysis was performed using the MetScape 3 App for Cytoscape (http://metscape.med.umich.edu). *Metscape is part of the National Institutes of Health-supported National Center for Integrative Biomedical Informatics (NCIBI) suite of tools, freely available at *http://metscape.ncibi.org*. It can be downloaded from *http://cytoscape.org* or installed *via* Cytoscape plugin manager.* Panel (**a**) urea metabolism linked to proline and ornithine metabolism; panel (**b**) branched chain amino acids metabolism. Node colors and shapes indicate different molecules.

We built an interaction network using the list of metabolites obtained to track the relations among metabolites and genes. We used MetScape (http://metscape.med.umich.edu from Cytoscape) which provides a bioinformatics framework for the interpretation and visualization of human metabolomics data^16^^.^ It analyzes metabolic networks using an internal database that integrates data from KEGG (Kyoto Encyclopaedia of Genes and Genomes) and EHMN (Edinburgh Human Metabolic Network). It allows to identify enriched pathways from expression profiling data, build and analyze the networks of genes and metabolites, and visualize changes in the gene/metabolite data^17^. We built the input list using the metabolites reported in Table 2. The pathways involved that emerged from metabolomics are reported in Fig. 3 showing the involvement of urea metabolism linked to proline and ornithine metabolism (panel a) and branched chain amino acids (panel b) metabolism.

## Discussion

**Figure 4:**
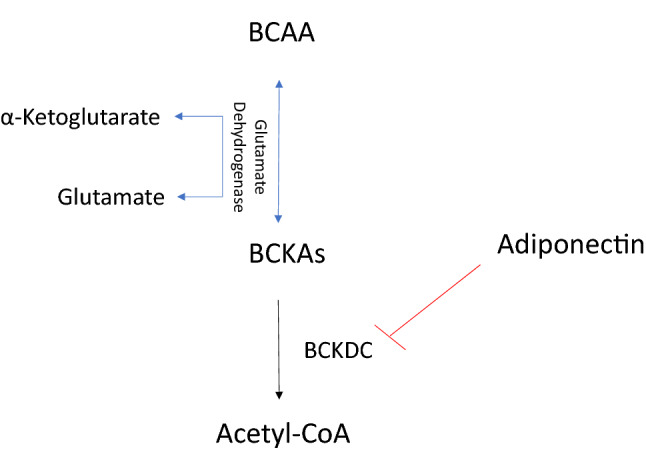
Inhibition by adiponectin on branched-chain amino acids catabolism [27]. Branched chain amino acids (BCAAs); Branched chain alpha keto acids (BCKAs); Branched-chain α-ketoacid dehydrogenase complex (BCKDC).

**Figure 5:**
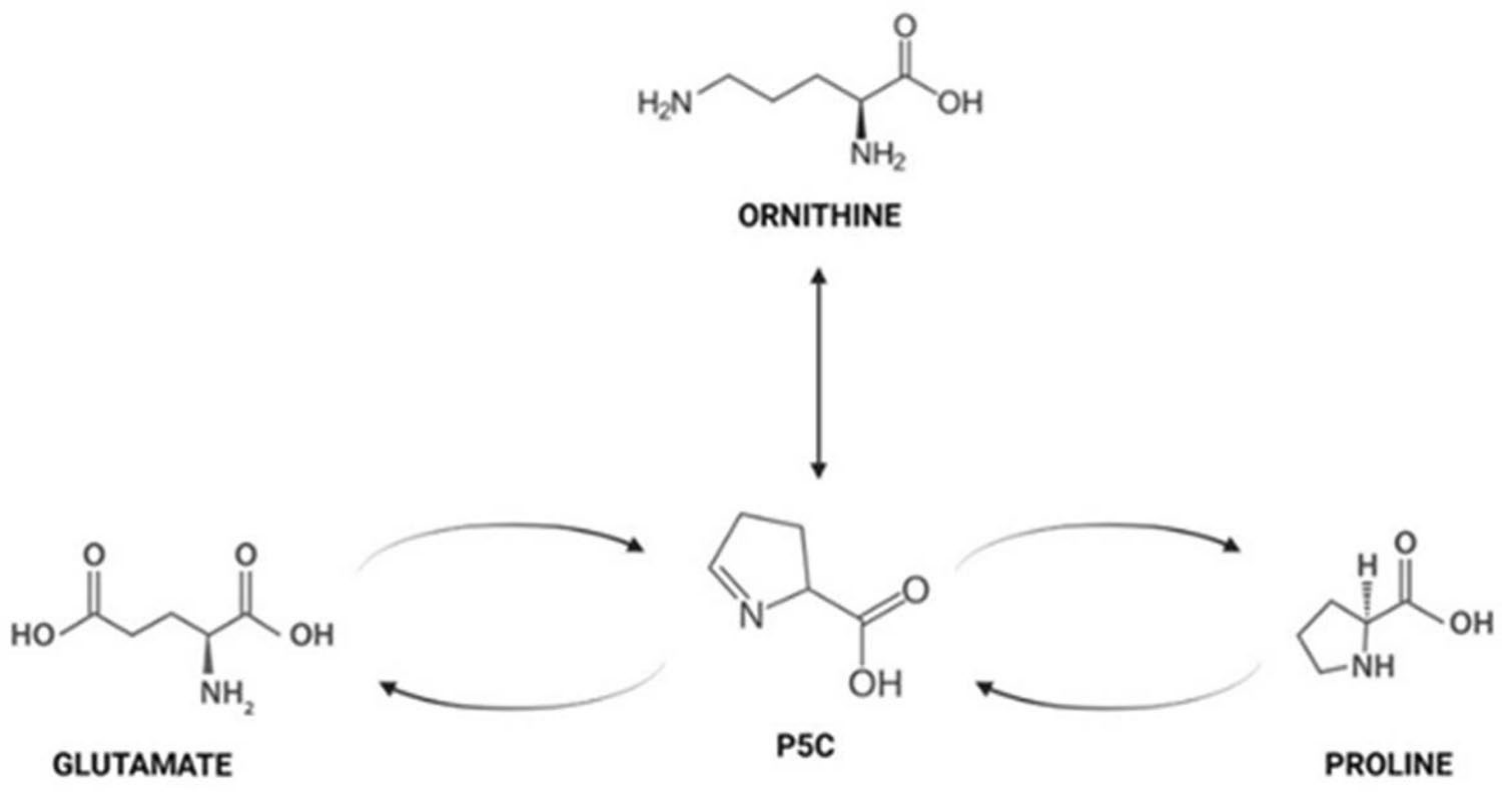
P5C cycle: Interconversion of proline to glutamate and ornithine; (P5C) pyr-roline-5-carboxylate. Figure was carried out by using BioRender (http://biorender.com).

Sportomics is the application of metabolomics in sports to investigate the metabolic effects of physical exercise on professional athletes and in this study metabolic profile and oxidative stress of plasma from elite soccer and basketball athletes were evaluated during training season to learn more about the prevailing metabolic pathways in two different sports and to monitor physical training in order to preserve and improve the athlete’s health. In soccer, power and strength greatly affects the game especially during sprinting and performing various ball skills^18^. Moreover, it is reported that soccer athletes show an increase in marker of muscle damage indicating muscle micro trauma and an increase in markers of inflammation^19^. As regards basketball match, which is 40–48 min long, athletes perform a combination of high-intensity actions such as sprinting, running, jumping, shuffling with low-intensity activities like walking^20^. The results obtained under our experimental conditions pointed out that in elite athletes with similar BMI, antioxidant capacity and adiponectin plasma levels show a significant reduction in plasma from soccer in comparison to basketball players. Moreover, soccer athletes show an increase in oxidative species in comparison with basketball players and healthy controls. We can hypothesize that these differences may be due to different duration and intensity of numerous game match during the competitive season^21,22^. In soccer players, we propose that the low level of adiponectin showed in our data, is not sufficient to carry out its prolonged anti-inflammatory action effect^23^. All these data suggested that soccer athletes are subjected to a continuous acute oxidative stress in comparison to basketball players and this confirm what reported by several authors that well-trained athletes present higher baseline adiponectin than non athletes^24^. However, data on the relationship among muscular fitness and adiponectin levels are few; nevertheless, Agostinis Sobrinho, suggests that adiponectin plasma levels is inversely associated with muscle strength^25^. In our experimental conditions, we found, in plasma from athletes, differences in isoleucine and valine, two of the branched-chain amino acids (BCAA), lactic acid and urea both involved in muscular metabolism. In plasma from basketball players, we observed an increase in isoleucine and valine indicating, as reported in literature, their mobilization from liver or muscle^26^. These amino acids in skeletal muscle promote glucose uptake and protein synthesis playing an important role during exercise especially in post-exercise recovery^27^. It is interesting to note that the amino acid proline shows a different trend in athletes respect to controls: in basketball, it does not significantly change while on soccer athletes its plasma level decreases. The metabolism of this amino acid involves the interconversion of proline to glutamate, an ammonia carrier generated in skeletal muscle during exercise^28^. We found in basketball players a decrease of glutamate level and we suppose that is due to supply ammonia to branched chain alpha keto acids (BCKAs) to generate BCAA increasing their plasma level in athlete as reported in Fig. 4. Moreover, we found an increase in proline and ornithine plasma level in basketball players and, as reported from Nowakowska, these two amino acids have an established role in defending against various stress^29^. In particular proline function is connected to the regulation of ROS homeostasis because its acts as a chaperone and stabilizes proteins, including antioxidant enzymes^30^. Proline is also converted in ornithine via pyrroline 5 carboxilic acid (P5C) Fig. 5 and the direct link of the proline P5C cycle to NADPH balance can have a deep impact on redox balance^31^. In our results, we found an increase in proline and ornithine plasma level and a reduction in glutamate in basketball players compared to soccer players suggesting that in these athletes the role of proline/ornithine metabolism, as reported in Fig. 3a, appears to be an important phenomenon in ROS regulation and redox homeostasis. In fact, in these athletes, we found a better redox homeostasis related with the higher antioxidant capacity and lower oxidative species production. Understanding the relationship among proline/ornithine/glutamate and ROS will affect cellular signalling and will improve human sport activity and performance. In addition, in basketball players we found an increase in plasma adiponectin level that is reported to inhibit, in skeletal muscle cells, the activity of branched-chain α-ketoacid dehydrogenase complex (BCKDC) which suppressed BCAA catabolism and leads to increase their plasma levels^28^. Our results can make us to speculate that in basketball the type of training and the type of game positively modify muscle metabolism, improving the adaptation to stress and inducing performance. Finally, our data indicate that urea levels decrease in athletes respect to healthy controls. Urea in professional athletes might be a signal of a strenuous training session and it could be considered as a recovery parameter^29^. Furthermore, several authors reported that urea value can be used to assess training load to improve sports performance as a low concentration indicates the need to increase exercise load levels^32,33^. The main limitation of our study, considered as a pilot study, is the low number of participants analyzed. Moreover, we plan to verify the effects of any single molecules identified in this study in human skeletal cells to understand metabolic pathways involved.

## Materials and methods

### Materials

Unless specified all reagents were obtained from Sigma (St. Louis, MO) except PVDF membrane (Millipore, Bedford, MA).

### Participants

A cohort of 30 male participants, 10 professional soccer players (S)10 professional basketball players (B) and 10 male students who did not practice habitual hard exercise as healthy controls (C), were enrolled in this study. The elite basketball athletes belonged to the Italian sport club “US AFFRICO-Firenze” while soccer players were recruited from the Italian sport club “Società Sportiva Arezzo”. Male controls were selected among the students of the degree course in Motor Sciences, Sport and Health of the Florence University. All enrolled athletes train regularly for more than 5 years. All participants were adults, selected according to age between 20 and 30 years old, of Caucasian ethnicity and they are all Italians, they completed a medical history and physical activity questionnaire in order to determine the eligibility. Dietary characteristics were evaluated using a questionnaire. None of them used antioxidant or nutritional supplements and they were selected because of non-smoking status, age and stable body weight. Each athlete follows a training program proposed by the coach and adapted to specific sports. The recruited players follow a training routine of five days per week, with sessions lasting 2 h per day. The training protocol involved technical and aerobic exercise. As far as strength training is concerned, the program for both players can be summarized as follows: (A) on shoulder muscles by performing the classic front and side lifts with weightlifting dumbbells: three sets of 12 repetitions; (B) work with the abdominal muscles, essential to maintain balance in every movement and to contribute to a good deadlift in jumps: three sets of 15 repetitions. For soccer players, this training program is supplemented by double under, effective for increasing the explosive strength of the legs and front squat using a barbell. Team coaches tracked day-to-day training data for each player and for each training session over the entire competition season^34^.

After the explanation of all experimental procedures, a written informed consent was provided prior to enrolment in the study that was conducted according to policy statement set forth in the Declaration of Helsinki The study was approved by the local Ethics Committee of the University of Florence, Italy (AM_Gsport 15840/CAM_BIO). During the study period, the trained subjects had their optimal body composition (ie, lowest fat mass and highest fat-free mass). Weight is measured to the nearest 0.1 kg and height to the nearest 0.5 cm. Body mass index (BMI) was calculated from the ratio of body weight (kg) to body height (m^2^). The same operator performs all measurements in athletes resting conditions, and biological samples are collected in morning, 48 h after a competition and 24 h after a training session. Capillary blood sample is picked up using a heparinized Microvette CB300 (Sarstedt AG and Co) from each participants. The blood sample was collected with capillary tip, holding the Microvette in a horizontal position (filling volume 300 µl). To obtain plasma the blood tubes were immediately centrifuged for ten minutes at 2000 × *g* using a table centrifuge. Plasma samples were stored at − 80 °C in freezer. The measurments were done on stored samples. The reduced invasiveness, the simplicity of execution and the lower cost led us to prefer the capillary collection to the venous, moreover small volumes of samples were sufficient to carry out all experiments.

### Plasma oxidative stress measurements

The d-ROMs test (diacron Reactive Oxygen Metabolite) and the BAP Test (Biological Antioxidant Potential) were used to determine the levels of reactive oxygen metabolites and the antioxidant capacity on plasma^32^. The biomarkers dROM and BAP were selected based on their long-term stability. In detail, d-ROM test is based on Fenton’s reaction in which in an acid buffer, plasma proteins release iron which induce the degradation of hydroperoxides into free alkoxyl and peroxy radicals. This free radicals react with the colourless N,N-diethyl-para-phenylendiamine (DEPPD) probe, which is converted into a stable coloured radical detectable at 505 nm. The BAP test provides a global antioxidant capacity of the sample, measured as its reducing potential against ferric ions. All analyses were carried out using a free radical analyzer system (FREE carpe diem—Diacron International srl) which included a spectrophotometric device reader and a thermostatically regulated minicentrifuge, and the measurement kits were optimized to the FREE Carpe Diem System, according to the manufacturer’s instructions^35^.

### Adiponectin western blot analysis

Plasma samples were clarified by centrifugation and the total protein contents were measured using Bradford assays after diluting the plasma samples suitably^36^ (1:50). An equal amount of each sample (10 μg of total proteins) was added to 4 × Laemmli buffer (0.5 M TrisHCl pH 6.8, 10% SDS, 20% glycerol, β-mercaptoethanol, 0.1% bromophenol blue) and boiled for 10 min. Samples were separated on 12% SDS/PAGE and transferred onto PVDF membrane using Trans-Blot Turbo Transfer System (BIO-RAD). Western blot was performed using a primary antibody (Acrp30 Santa Cruz) diluted 1:1000 in 2% milk. After incubation with horseradish peroxidase (HRP)-conjugated antimouse IgG (1:10,000) (Santa Cruz Laboratories), immune complexes were detected with the enhanced chemiluminescence (ECL) detection system (GE Healthcare) and by Amersham Imager 600 (GE Healthcare). For quantification, the blot was subjected to densitometry analysis using ImageJ program. The intensity of the immunostained bands was normalized with the total protein intensities measured by Coomassie brilliant blue R-250 from the same PVDF membrane blot as previously reported^35^.

### Gas Chromatography—Mass Spectrometry (GC–MS) analysis of plasma samples

Metabolite analyses were performed on three pools of plasma sample, one for each group (basketball, soccer and control group). Pooling samples is not a perfect option, however, it can be of help when few samples are available and this jeopardize biomarker discovery approaches^36^. Moreover the pooled plasma samples strategy to reduce individual differences of inter-individual variation in metabolomic and proteomic studies and enhance the confidence of the study^37^. Each pool was made taking the same volume of plasma from each participant in order to reduce intra individual variability of metabolites levels. The GC–MS analysis was performed on 100 µl of plasma as previously reported^35^. The injection volume was 1 µL and a split ratio of 1:10 was used. The MassHunter data processing tool (Agilent) was used to obtain a global metabolic profiling^35^. Metabolite identification was performed at level 1 as proposed by the metabolomics standards initiative^38^ using retention index and mass spectrum.as the two independent and orthogonal data required for identification. As reference library of compounds, we used the Fiehn Metabolomics RTL library (Agilent G1676AA) that was obtained with the same instrument used for our analyses^39^.

### Statistical analysis

Data are presented as means + /− standard deviation (SD) from at least three experiments. The univariate data analysis was performed as one-way ANOVA and the differentially expressed spots (ANOVA p-value < 0.05) were subsequently analysed by Tukey’s multiple comparisons test using GraphPad Prism v6.0 software to find out the significant differences between groups. Significance was defined as p-value < 0.05.

### Ethical approval

The study was conducted in accordance with the Declaration of Helsinki, and approved by the Comitato Etico Regionale per la Sperimentazione Clinica della Regione Toscana Sezione: AREA VASTA CENTRO, Italy (AM_Gsport 15840/CAM_BIO—24th february 2020).

### Informed consent

A written informed consent was provided prior to enrolment in the study.

## Conclusions

Our results highlight that soccer in comparison with basketball athletes, are subject to higher continuous oxidative stress associated with possible inflammation, probably due to the duration and intensity of the physical effort required in this discipline. The significant increase of BCAA plasma level in basketball players, the contemporary decrease in glutamate and the increase in adiponectin could suggest the inhibition of BCAA catabolism and their stimulation effect in glucose uptake and catabolism. We suggest that the high level of adiponectin measure in basketball players could be able to counteract the local inflammatory effects of exercise loads. Moreover, the proline/ornithine/glutamate pathway seems to play a role in muscle metabolism linked to maintenance of proper NAD + /NADH levels in the cytosol connected to the regulation of ROS homeostasis. In order to understand the role of these plasma modifications, future studies will be performed in skeletal muscle cells. Our future experiments will use the molecules identified in this work to stimulate muscle cells to analyze metabolic pathways involved in cellular response. Moreover, it will be interesting to analyze which molecules will be released from these cells in order to understand the signaling among trained skeletal muscle and tissues.

## Data Availability

The datasets used and/or analyzed during the current study are available from the corresponding author on request.
